# The Values Supporting the Creativity of Employees

**DOI:** 10.3389/fpsyg.2021.805153

**Published:** 2022-02-03

**Authors:** Miluše Balková, Pavla Lejsková, Lenka Ližbetinová

**Affiliations:** ^1^Department of Human Resource Management (HRM), Faculty of Corporate Strategy, Institute of Technology and Business in Ceské Budejovice, Ceské Budejovice, Czechia; ^2^Department of Transport Management, Marketing and Logistics, Faculty of Transport Engineering, University of Pardubice, Pardubice, Czechia

**Keywords:** human resource management, creativity, company values, employees, company size, innovation, HRM 4.0

## Abstract

In Industry 4.0 completely new production worlds are emerging, where robots are becoming a key element and where common human skill activities and thinking are commonly surpassed. The growing degree of automation and the interconnection of the digital and the real-world create an environment that requires a set of interdisciplinary skills. For the sustainability of enterprises in this environment, human creativity acquires an irreplaceable role. The aim is to compare the application of selected values in corporate culture, which creates a space for increasing the creativity of employees from the perspective of different sizes of enterprises. The research sample consists of 1,716 companies of the Czech and Slovakia that participated in the questionnaire survey. The results of the independent *T*-test confirmed significant differences in the applied values between the countries being compared. Differences in terms of business size have been further explored through the ANOVA test and the Tukey HSD test. The results subsequently confirmed that the values: work meaningfulness, passion for work, and trust are applied more significantly to microenterprises in both the countries, creating an environment that supports creativity.

## Introduction

One of the prerequisites for business success in a rapidly changing world is the ability to respond to changes in technology, human resource management, or the use of new business models. Digitization is becoming an integral part of sustainable business life. In Industry 4.0, completely new production worlds are emerging, where robots are becoming a key element and where common human skill activities and thinking are commonly surpassed. The growing degree of automation and the interconnection of the digital and real-world create an environment that requires a set of interdisciplinary skills. These trends are observable in various areas of the economy (Davidson, 2020; Duft and Durana, 2020; Lazaroiu et al., 2020). For the sustainability of enterprises in this environment, human creativity acquires an irreplaceable role. Innovation and open flexible thinking about the necessary changes can give operators the necessary competitive advantage to overcome a turbulent period of change (Connolly-Barker et al., 2020; Kovacova et al., 2020; Kovacova and Lewis, 2021; Hawkins, 2021).

Creative thinking can be understood as an exclusively “human” skill that no artificial intelligence can replace. The originality of the ideas that people are able to bring differentiates products or services from the competition. Supporting and using creativity in the company is a great challenge for managers at all levels (Kucharcikova et al., 2021).

The company’s ability to support innovation and creativity as inimitable intangible resources is a key element of competitive advantage (Scott, 1995; Stehel et al., 2021). The art of offering innovative products needs creative employees who are motivated (Hitka et al., 2018) to develop their creative potential and to generate creative ideas (Jovčić et al., 2019). Lack of motivation (Bartakova et al., 2017; Hitka et al., 2021) can lead talented workers to avoid job opportunities and make little effort to improve their performance. Inefficient use of employee talent (Gottwald et al., 2015) is considered a waste of resources that leads to a lack of creativity, reduced market share, competitive advantage (Milenković et al., 2015; Dobrodolac et al., 2016), and reduced customer satisfaction (Khalil et al., 2017). The successful sustainable development of the company includes the synergy of quality management (Nedeliakova et al., 2016), technology, and well-motivated employees (Hitka et al., 2019), whose common basis are shared and applied corporate values (Lorincová et al., 2018; Song et al., 2020).

Published studies focus on finding a link between values and increased creativity, but there is a lack of more comprehensive research on the current state of application of values in companies, according to selected factors. This paper helps to fill this knowledge gap by mapping the current situation in the Czechia and Slovakia. This study deals with how often companies in the Czechia and Slovakia apply selected values that are important in encouraging employees to engage in a creative behavior. A comparison of the application of values according to the size of enterprises and a comparison of both countries may provide a new perspective on the approach of enterprises on the sustainable development at the time of the advent of Industry 4.0. The aim is to compare the application of selected values into corporate culture, which creates space for increasing the creativity of employees from the perspective of different sizes of enterprises.

## Literature Review

The pressure for constant innovation supported by digital transformation and the use of elements of artificial intelligence (Nedeliaková and Panák, 2015; Nývlt and Juhásová Šenitková, 2018) increases the need for flexible business behavior and the support of creativity in its employees (Hyršlová et al., 2007; Jaros et al., 2014; Kampf et al., 2018; Lazarevic et al., 2020). In the conditions of Industry 4.0, the effective use of creativity by employees is predicted by many factors (Blštáková et al., 2020; Stacho et al., 2020; Stachová et al., 2020). It is mainly the personality of the employee and his internal or external motivation related to the application of shared values in the company (Fila et al., 2020; Szeiner et al., 2020; Gódány et al., 2021). Locke and Latham (1990) hypothesized that corporate culture influences employee behavior through shared applied corporate values. Kirkman and Shapiro (2001) found that differences in applied corporate values affect employees’ access to their job duties and responsibilities, which pointed out the link between corporate values and individual work performance. The applied company values are closely related to the work motivation of employees. Kaplan et al. (2009) pointed out that employees with different values have different approaches to work tasks and goals and also to different performances in creativity. By applying common corporate values, companies can influence the motivation and work behavior of their employees (Kuptcova et al., 2016; Nedeliakova et al., 2018). In this sense, corporate values should be considered as an important factor in examining sustainable development by supporting the stimulation of employee creativity.

In many studies, the creative personality is associated with such traits as independence, entrepreneurship, radicality, and openness to experience (Lubart et al., 2015; Martin et al., 2015; Potkany et al., 2021). Wang et al. (2021) used a questionnaire survey to show that there is a relationship between an individual’s creativity, self-confidence, and set goals. Through a questionnaire survey, Zhang H. et al. (2021) revealed that the ability to acquire new knowledge and the willingness to share that knowledge positively affects social curiosity, sensitivity to deprivation, and the joy of discovery. According to their outputs, the willingness to pass on knowledge has a direct impact on the level of creativity in the company. The Baldé et al. (2018) demonstrated the connection between mutual trust in the team in the workplace and internal motivation, which encourages employees in the formation of individual knowledge and creativity and a willingness to share knowledge mutually.

The correlation between the application of shared values in the company and the innovative behavior of employees was examined by Yeh-Yun Lin and Liu (2012). They show that innovative employee behavior mainly affects organizational encouragement, encouragement by supervision support of work groups, sufficient resources, and meaningfulness of work. Applying common values, creative behavior of employees and the willingness to share knowledge and new ideas are closely related to the chosen way of motivation (Ručková et al., 2018; Doktorová and Varečková, 2021). Zhang Y. et al. (2021) confirmed through field studies the importance of convergent support for the internal motivation mechanism, which is based on a positive relationship between the effect of reward on individual performance and employee creativity. These are individuals with a high vertical (but not horizontal) team orientation. According to Zhang Y. et al. (2021), the understanding of how external rewards affect creativity leads to emphasizing the importance of taking into account individual differences in cultural values. In contrast, research conducted using a questionnaire survey in the energy industry in Vietnam did not show (Vu et al., 2021) a statistically significant relationship between intrinsic motivation at the experiential level and employee creativity. In addition, external incentives related to direct rewards have not been identified as a decisive factor in creating innovative proposals. Shalley et al. (2004) suggested that there is little agreement among scientists about the likely direction of the effects of conditional rewards on an individual’s expressed creativity, but that it depends more on shared values. Oyedele (2013), in his study, identified seven value dimensions that can be demotivating in relation to creativity. These include organizational injustice, stress, poor coordination, poor interpersonal relationships, career decline, negative management behavior, and poor organizational culture.

The creative behavior of employees is positively influenced by support in the form of further education and regular evaluation of performance and recognition (Gong et al., 2009; Mura et al., 2019). The creativity of employees in the workplace can also be influenced by work experience gained during working life. González-González and García-Almeida (2021) examined work experience and engagement to seek opportunities for improvement and innovation through regression analysis of the survey data. They came to conclude that work experience, engagement, and passion serve as an important input for identifying opportunities for improvement and for generating new ideas. Muñoz-Pascual et al. (2021) confirmed through quantitative research and data analysis that knowledge, motivation, and relationships have a positive and significant influence on creativity, which allows more ideas to be developed. They found that factors associated with “tacit knowledge” (experience, skills, training, and practical courses) have a major impact on the development of new ideas. They revealed that external motivation factors, such as group incentives, make a significant contribution to the development of creativity and sustainable innovation. According to the authors, the factors associated with intrinsic motivation (satisfaction, determination, responsibility, Identification, and consideration of problems) have a great impact on the development of new ideas and sustainable innovation. These motivations activate the applied values in the company such as open communication, autonomy, responsibility, and passion.

Görzen (2021) addressed in his study whether the creativity of employees is stimulated by the importance of the assigned task. He found out through a field experiment that higher importance of the task has no positive effect on the quantity or creativity of the output. Creativity is influenced by a commitment to the success of the enterprise, passion for the cause, and feedback from the management (recognition). In contrast, Pirola-Merlo and Mann (2021) argue that employees working in a team are more creative if they know the meaning and meaningfulness of the tasks.

The intrinsic motivation for innovative and creative behavior of managers was examined by Özdemir et al. (2021). They confirmed that the level of innovative and creative behavior of managers is significantly influenced by values such as support, emphasis on health, commitment, subjective poverty, and risk behavior. A new factor that can influence the creativity of employees is the application of the values of open communication, support, and trust through the use of Internet applications. Zhang H. et al. (2021) investigated the effect of using WeChat at work on employee creativity. Using empirical research and the use of a structural equation model, they revealed that the excessive use of WeChat directly promotes creativity (open communication and meaningfulness) and indirectly improves creativity through knowledge sharing (collaboration and recognition). Ozer and Zhang (2021), through repeated questionnaire surveys, have shown that employees who need to connect with other employees gain high-quality interpersonal relationships with their coworkers and they also show a high level of creativity due to the use of social networks to communicate between employees.

Based on the published findings, it was possible to compile a list of values that are related to the support of employee creativity and increasing innovative thinking. There is a possibility to include (among the values supporting creativity):

• A1–the meaningfulness of work (employees know the meaning of their work);
• A2–engagement according to;
• A3–enthusiasm resp. passion;
• A4–cooperation;
• A5–recognition (public, personal);
• A6–open communication;
• A7–support;
• A8–autonomy;
• A9– emphasis on health;
• A10–trust;
• A11–responsibility (inner sense of responsibility).

The need for a creative approach to innovation in a changing environment due to changes caused by, for example, Industry 4.0 or the current COVID crisis, opens the research question: To what extent do companies currently apply these values? The assumption of different possibilities according to the size of the company also offers another research question: Does this application of values differ in terms of the size of companies?

## Materials and Methods

The aim was to compare the application of selected values in corporate culture and create space for increasing the creativity of employees in the perspective of different sizes of enterprises. The questionnaire survey was used to meet the aim of the study, which determined the degree of application or the importance of values in the company, and which also has an effect on the support of employee creativity. The survey took place in the period from spring 2020 to spring 2021. Employees responsible for the HR area in companies were addressed within the Czech and Slovakia (proportional selection according to size). There were 1,716 enterprises that participated in the questionnaire survey of which 608 enterprises were from the Czechia and 1,108 were from Slovakia (see Table 1). The basic set consists of 2,932,963 business entities in the Czechia (CZE) (95.45% micro, 3.45% small, 0.91% medium, and 0.19% large enterprises) and in the Slovakia (SVK). In terms of the population according to the Statistical Office, there were 2,932,963 Czech business entities in 2020 (95.45% micro, 3.45% small, 0.91% medium, and 0.19% large enterprises), and in Slovak business entities there were 635,876 (98.4% of micro and small enterprises).

**TABLE 1:** Composition of the research file.

State	Enterprise size	Frequency	Percentage
Czechia (CZE)	Micro	108	17.8
	Small	143	23.5
	Medium	159	26.2
	Large	198	32.6
	Total for the CZE	608	100
Slovakia (SVK)	Micro	285	25.7
	Small	231	20.8
	Medium	235	21.2
	Large	357	32.2
	Total for the SVK	1108	100
Total for both countries	Micro	393	22.9
	Small	374	21.8
	Medium	394	23.0
	Large	555	32.3
	Total	1716	100

*Source: authors.*

The persons responsible for the HR area in the company evaluated the extent to which the examined values are applied within their personnel strategy. The degree of importance and the current application of values were evaluated on a rating scale from 1 to 5, where 1 means we do not apply and 5 we fully apply.

The assessed values (which are considered significant for the application of creativity in the enterprise) were set according to current research studies about employees’ creativity. The compiled list of values consists from: A1: the meaningfulness of work (employees know the meaning of their work) (Yeh-Yun Lin and Liu, 2012; Pirola-Merlo and Mann, 2021; Zhang H. et al., 2021); A2: engagement according to González-González and García-Almeida (2021) and Özdemir et al. (2021); A3: enthusiasm resp. passion (González-González and García-Almeida, 2021; Görzen, 2021; Muñoz-Pascual et al., 2021); A4: cooperation (Muñoz-Pascual et al., 2021; Ozer and Zhang, 2021; Zhang H. et al., 2021; Zhang Y. et al., 2021); A5: recognition (public, personal) (Gong et al., 2009; Oyedele, 2013; Görzen, 2021; Zhang H. et al., 2021); A6: open communication (Oyedele, 2013; González-González and García-Almeida, 2021; Muñoz-Pascual et al., 2021; Zhang H. et al., 2021); A7: support (Gong et al., 2009; Yeh-Yun Lin and Liu, 2012; Özdemir et al., 2021); A8: autonomy (Muñoz-Pascual et al., 2021; Wang et al., 2021); A9: emphasis on health (Oyedele, 2013; Özdemir et al., 2021); A10: trust (Baldé et al., 2018); A11: responsibility (inner sense of responsibility) (Görzen, 2021; Muñoz-Pascual et al., 2021). Questionnaire responses were analyzed using SPSS Statistics 26. Enterprises were analyzed by size and country using descriptive statistics and also using tests to determine differences in mean values, independent Student’s *t*-test, parametric ANOVA test, and Tukey’s HSD test. All these tests are solved at a significance level of 5% and 1% (two-tailed).

The following research questions have been determined: To what extent do companies currently apply these values and is there a difference in the application of the assessed values from the point of view of the surveyed countries? Is this application of values different in terms of the size of companies? The results should answer the following research questions:

• Research hypothesis RH1: There is no difference between the two countries in the use of the assessed applied values.
• Research hypothesis RH2: There is no connection between the size of the company and the applied values influencing the creativity of employees.

## Results

**FIGURE 1:**
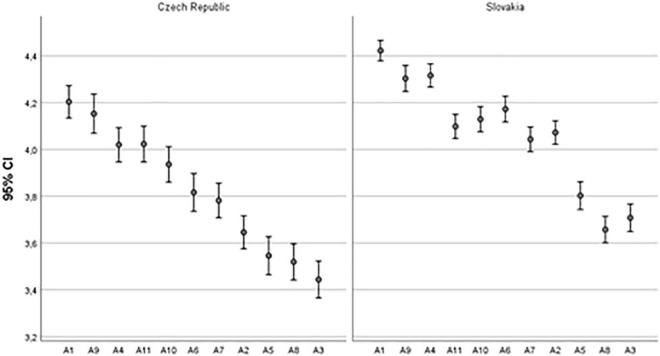
Comparison of the application of selected values in CZE and SVK. Source: authors.

Based on the evaluation of individual values in both the countries (Figure 1), the values A1 (the meaningfulness of work, average for both countries together, average 4.51) and A9 (emphasis on health, 4.47) are the most applied. On the other hand, the least applied are values A3 (enthusiasm or passion, 3.92) and A8 (autonomy, 3.94). In enterprises of the Czechia, the values A1 (meaningfulness, 4.39) and A9 (emphasis on health, 4.36) are the most applied and the least applied is A3 (enthusiasm and passion, 3.68). According to the outputs in Slovakia, the most applied values are A1 (meaningfulness, 4.58) and A9 (emphasis on health, 4.53), and the least implemented value is A8 (autonomy, 4.01).

The differences between Czech and Slovak companies in the approach to the application of selected values supporting the creative behavior of employees are shown in Figure 1. According to it, these values are applied more in Slovakia. The Student’s *t*-test verified if there are statistically significant differences between the countries (Table 2). The results of Table 2 show that there are significant differences (from the point of view of the country at the level of significance of 5%) for all the monitored values, except the value of A11, which is responsibility (internal sense of responsibility). It can therefore be stated that H1 is not rejected at the level of significance of 5% because for one of the monitored values no significant difference was determined according to the affiliation of the company to the state.

**TABLE 2:** Independent samples test for differences in values between states.

Number of the value	Levene’s Test		*t*-test for equality of means				
	F	Sig.	Df	Sig. (2-tailed)	Std. error difference	95% confidence interval	
						Lower	Upper
**A1**	9.239	**0.002**	1715	0.000	0.040	−0.297	−0.141
			1097.726	**0.000**	0.042	−0.300	−0.137
**A2**	9.360	**0.002**	1715	0.000	0.044	−0.512	−0.341
			1202.579	**0.000**	0.044	−0.514	−0.340
**A3**	0.001	0.974	1715	**0.000**	0.050	−0.363	−0.166
			1260.965	0.000	0.050	−0.363	−0.166
**A4**	1.691	0.194	1715	**0.000**	0.044	−0.382	−0.210
			1158.284	0.000	0.045	−0.385	−0.208
**A5**	2.461	0.117	1715	**0.000**	0.051	−0.357	−0.157
			1236.253	0.000	0.051	−0.358	−0.157
**A6**	4.363	**0.037**	1715	0.000	0.049	−0.452	−0.261
			1168.808	**0.000**	0.050	−0.454	−0.259
**A7**	5.691	**0.017**	1715	0.000	0.046	−0.352	−0.172
			1213.808	**0.000**	0.046	−0.353	−0.171
**A8**	0.001	0.981	1715	**0.004**	0.049	−0.233	−0.043
			1237.919	0.005	0.049	−0.234	−0.043
**A9**	6.050	**0.014**	1715	0.002	0.050	−0.248	−0.053
			1144.127	**0.003**	0.051	−0.251	−0.050
**A10**	0.001	0.975	1715	**0.000**	0.047	−0.285	−0.101
			1204.761	0.000	0.047	−0.286	−0.100
A11	0.979	0.323	1715	0.100	0.046	−0.165	0.014
			1156.132	0.110	0.047	−0.168	0.017

*Significant differences at the 5% significance level are highlighted in bold.*

*Source: authors.*

### Values According to the Size of the Company in the Czechia

Another subject of research was how the application of the monitored values depends on the size of the enterprise in the Czech and Slovakia. According to the results of the arithmetic averages (Table 3), the values that can contribute to the creativity of employees in the Czech environment are the most used by microenterprises, except for the value A4, cooperation and A9, emphasis of health. The value A4 is most often applied by small companies and the value A9 is more applied by large companies. The ANOVA test (at a significance level of 5%) confirmed that the size of a company has a statistically significant effect on the application of the values A1, meaningfulness, A3, enthusiasm, A9, emphasis on health, and A10 confidence in the Czechia. These values were further investigated using the test Tukey HSD (Table 4) at a significance level of 5%. This test determined the specific relationships of differences in the case of A1, A3, and A10 between a microenterprise, where the values are more applied compared to a medium-sized enterprise, and in the case of A3 and A10 also between a micro and a large enterprise. For A9, there is a significant difference between a small enterprise (where the value is least applied) compared to a medium and large enterprise.

**TABLE 3:** Arithmetic averages by size of enterprise, CZE.

Size of enterprise	A1	A2	A3	A4	A5	A6	A7	A8	A9	A10	A11
Micro-enterprise	**4.54**	**4.15**	**4.01**	4.28	**3.99**	**4.14**	**4.22**	**3.96**	4.32	**4.44**	**4.42**
Small-enterprise	4.42	3.92	3.71	**4.31**	3.58	4.08	3.94	3.77	4.10	4.20	4.33
Middle-enterprise	4.23	3.84	3.54	4.21	3.78	3.83	4.03	3.61	4.42	4.10	4.11
Large-enterprise	4.42	3.92	3.57	4.19	3.80	4.09	4.11	3.93	**4.52**	4.05	4.20
Total	4.39	3.94	3.68	4.24	3.77	4.03	4.07	3.81	4.36	4.17	4.25

*The largest rate of application of the observed values within the size of enterprises is highlighted in bold.*

*Source: authors.*

**TABLE 4:** Tukey’s test: comparison of the application of motivational factors according to the size of the company in CZE, and the resulting *p*-values.

Dependent variable	Anova test *p*-value	Enterprise	*p*-value/size of enterprise			
			Micro	Small	Middle	Large
A1–meaningfulness	**0.025**	**Micro**		0.703	**0.018**	0.625
		Small	0.703		0.205	1.000
		**Middle**	**0.018**	0.205		0.167
		Large	0.625	1.000	0.167	
A3–enthusiasm	**0.028**	**Micro**		0.315	**0.033**	**0.042**
		Small	0.315		0.739	0.823
		**Middle**	**0.033**	0.739		0.997
		**Large**	**0.042**	0.823	0.997	
A9– emphasis of health	**0.005**	Micro		0.426	0.891	0.431
		**Small**	0.426		**0.060**	**0.003**
		**Middle**	0.891	**0.060**		0.811
		**Large**	0.431	**0.003**	0.811	
A10–trust	**0.010**	**Micro**		0.220	**0.034**	**0.007**
		Small	0.220		0.847	0.553
		**Middle**	**0.034**	0.847		0.962
		**Large**	**0.007**	0.553	0.962	

*Significant differences at the 5% significance level are highlighted in bold.*

*Source: authors.*

### Values According to the Size of the Company in the Slovakia

In Slovakia, these values are most often applied by microenterprises, with the exception of A9, emphasis on health, which is the most applied in medium and large enterprises (see Table 5), similar to the Czechia. ANOVA test (at the level of significance 5%) proved (Table 6) that in Slovakia the size of the company has a statistically significant effect on the application of values A1–A7 (A1, meaningfulness of work; A2, commitment; A3, inflammation; A4, cooperation; A5, recognition; A6, open communication; and A7, support) and A10–A11 (A10, trust; A11, responsibility). The relationship between the size of the company for the application of the values A8 (responsibility) and A9 (emphasis on health) in the corporate culture proved to be insignificant.

**TABLE 5:** Arithmetic averages by size of enterprise–SVK.

Size of enterprise	A1	A2	A3	A4	A5	A6	A7	A8	A9	A10	A11
Micro-enterprise	**4.67**	**4.44**	**4.28**	**4.62**	**4.17**	**4.59**	**4.46**	**4.12**	4.45	**4.58**	**4.54**
Small-enterprise	4.60	4.30	4.08	4.50	4.13	4.39	4.30	4.05	4.49	4.36	4.36
Middle-enterprise	4.43	4.26	3.89	4.41	3.87	4.24	4.24	3.92	**4.58**	4.29	4.29
Large-enterprise	4.59	4.36	3.91	4.53	4.09	4.31	4.29	3.95	**4.58**	4.22	4.27
Total	4.58	4.35	4.04	4.52	4.07	4.39	4.33	4.01	4.53	4.36	4.37

*The largest rate of application of the observed values within the size of enterprises is highlighted in bold.*

*Source: authors.*

**TABLE 6:** Tukey’s test: comparison of the application of motivational factors according to the size of the company in CZE, and the resulting *p*-values.

Dependent variable	Anova test *p*-value	Enterprise	*p*-value/size of enterprise			
			Micro	Small	Middle	Large
A1–meaningfulness	**0.000**	**Micro**		0.598	**0.000**	0.438
		**Small**	0.598		**0.027**	0.999
		**Middle**	**0.000**	**0.027**		**0.018**
		**Large**	0.438	0.999	**0.018**	
A2–engagement	**0.047**	**Micro**		0.175	**0.045**	0.552
		Small	0.175		0.949	0.806
		**Middle**	**0.045**	0.949		0.440
		Large	0.552	0.806	0.440	
A3–enthusiasm	**0.001**	**Micro**		0.268	**0.003**	**0.002**
		Small	0.268		0.392	0.423
		**Middle**	**0.003**	0.392		0.996
		**Large**	**0.002**	0.423	0.996	
A4–cooperation	**0.015**	**Micro**		0.297	**0.008**	0.444
		Small	0.297		0.498	0.973
		**Middle**	**0.008**	0.498		0.202
		Large	0.444	0.973	0.202	
A5–recognition	**0.022**	**Micro**		0.986	**0.020**	0.873
		Small	0.986		0.083	0.985
		**Middle**	**0.020**	0.083		0.110
		Large	0.873	0.985	0.110	
A6–open communication	**0.000**	**Micro**		0.052	**0.000**	**0.001**
		Small	0.052		0.323	0.780
		**Middle**	**0.000**	0.323		0.802
		**Large**	**0.001**	0.780	0.802	
A7–support	**0.028**	**Micro**		0.187	**0.035**	0.072
		Small	0.187		0.895	0.998
		**Middle**	**0.035**	0.895		0.936
		Large	0.072	0.998	0.936	
A10–trust	**0.000**	**Micro**		**0.025**	**0.002**	**0.000**
		**Small**	**0.025**		0.823	0.189
		**Middle**	**0.002**	0.823		0.756
		**Large**	**0.000**	0.189	0.756	
A11–responsibility	**0.000**	**Micro**		0.064	**0.004**	**0.000**
		Small	0.064		0.817	0.629
		**Middle**	**0.004**	0.817		0.996
		**Large**	**0.000**	0.629	0.996	

*Significant differences at the 5% significance level are highlighted in bold.*

*Source: authors.*

The Tukey HSD test proved closer relations applied values with the size of the enterprise (at a significance level of 5%). The statistically significant differences were identified between the microenterprises (where the value was more applicable) compared to the medium and large enterprises in the case of values A3, A6, and A11 (Table 6). The value A10 is differently applied between microenterprises (where it is applied more) compared to other size categories of enterprises. In the case of values A2, A4, A5, and A7, the significant difference was determined between the microenterprises and the medium-sized enterprises. Also, the value A1 (meaningfulness) is applied significantly less in medium-sized enterprises than in other size categories.

The research hypothesis RH1 was not confirmed (at the significance level of 1%) because there were statistically significant differences that were identified in the use of individual values (in the case of all assessed values except A11, responsibility) by countries within the Czechia and Slovakia (Table 2 and Figure 1). Similarly, the research hypothesis RH2 was not confirmed because there were statistically significant differences in the application rate of the assessed values due to the size of an enterprise, in the bout of countries. The ANOVA a HSD Tukey test revealed differences in the application of the four values (A1, meaningfulness; A3, enthusiasm; A9, emphasis of health, and A10, trust) supporting creativity between the different sizes of companies in the Czechia, and of the nine assessed values (A1, meaningfulness; A2, engagement; A3, enthusiasm; A4, cooperation; A5, recognition; A6, open communication; A7, support; A10, trust; and A11, responsibility) in the case of Slovakia.

## Discussion

### Comparison of Applied Values Depending on the Country

The results of the Student’s *t*-test showed that the application of values that can contribute to the creativity of the employees in Slovak and Czech enterprises is different. Only the value of A11 (responsibility) is applied in the same way within the compared countries. According to the results of the comparison of applied values, currently, the greatest emphasis is placed on the meaningfulness of work (A1) in both the countries. Therefore, employees are acquainted with the importance of their work for the company with an emphasis on health and cooperation. Values like trust, responsibility, and open communication are important for Czech enterprises. Within all companies, the value of A9 (an emphasis on health’) is very often applied, which should contribute to the feeling of a safe environment in the workplace and thus encourage creative behavior. This value was the most often applied in medium and large companies. The third most commonly applied value is A4 (a collaboration), which is most commonly used in small enterprises.

### Finding the Difference in the Use of the Assessed Values According to the Size of the Enterprise in Individual Countries

The Tukey test showed differences in the approach to the application of individual values, which shows that there are differences in the approach according to company size: for A1 values, meaningfulness between micro and medium enterprises, for the application of A3 values, enthusiasm; and A10, trust between microenterprises and medium and large enterprises in the Czechia. There is also a significant difference in the application of the A9 value, the emphasis on health, between small and large enterprises.

In Slovakia, there is a statistically significant difference in the application by size for almost all values. In particular, there is a different approach to A1 value, meaningfulness between micro, small, and medium-sized enterprises, and also between medium and large enterprises. The application of the values A2, engagement; A4, cooperation; A5, recognition; and A7, support, shows a statistically significant difference between micro and medium-sized enterprises. The values A3, ignition; A6, open communication; and A11, responsibility, show a different approach of micro, medium, and large enterprises. Then, the application of the value of A10, trust shows the difference between all types of companies. No statistically significant difference was found for the value of A8, autonomy and A9, emphasis on health.

In the Czechia, there is less connection between the size of the company and the application of individually selected values than in Slovakia. In Slovakia, all the selected values were applied more often and their point evaluation ranges from 4 to 5 points for all company sizes. In the Czechia, some values range from 3 to 4 points (A3-enthusiasm, A5-recognition, and A8-autonomy) and in some cases, respondents could not assess whether values were applied in their enterprises. Therefore, it can be concluded that in Czech companies employees are less motivated to behave creatively than in Slovakia.

## Conclusion

The constant development of today’s globalized world and Industry 4.0 creates pressure for the constant search for new practices, Technologies, and managerial skills. There is an ever-increasing demand for innovative and creative behavior of companies. The corporate environment and shared values that motivate employees to behave creatively can contribute to success. For the purposes of this study, company values were selected, which are most often mentioned in professional studies in connection with creative behavior [meaningfulness of work, commitment, enthusiasm and joy of work, cooperation, recognition (public, personal), open communication, support, autonomy, emphasis on health, trust, responsibility (inner sense of responsibility)]. The aim of this study was to compare Czech and Slovak companies in the application of selected values that stimulate the creativity of the employees. Companies were compared on the basis of their size. Both of them belong to countries with a rich European culture, and both have a similarly developed industry, a modern social system, and similar customs.

This contribution examined the questions: Is there a connection between the size of the enterprises and the assessed types of HR values that increase their creativity? Is there a difference in the use of the types of values under consideration between the two countries?

In Czech and Slovak companies, the values are mostly applied based on the knowledge of the meaningfulness of work and emphasis on a safe working environment. Within the Czechia, this application is used by values through enthusiasm (joy and enthusiasm for work) and values through autonomy, i.e., independence at work within Slovakia. In both countries, microenterprises are the most motivating creativity, which in Slovakia can be expected to increase the overall motivation for creativity than in the Czechia. The connection between the frequencies of the application of selected values and the size of the company was proved. In Slovakia, there are significant statistical differences between the size of the company and the frequency of application of the selected values. From the results of both the countries, it can be concluded that in small and microenterprises there is more room to create a creative corporate culture, which is based on interpersonal relationships and shared values. These values are mainly knowledge of the meaningfulness of work, communication, passion, and support. The values associated with the organization of work, such as creating a safe working environment, cooperation, and responsibility, are gaining importance with the growth of the business entity. Based on the above facts, it is possible to recommend the management of large companies to incorporate more values associated with interpersonal communication into the corporate culture, e.g., through working groups or departments, and thus to strengthen their position in the competitive environment. This study can serve as a basis for further research in the field of creative management, human resources, and further sustainable development. The presented research provides an overview of the application of values in companies in selected countries, and thus complements the knowledge gap of the issue of currently created conditions to support the creativity of employees for services. From a practical point of view, it provides instructions resp. a recommendation as to which value needs to be applied in a sophisticated way.

## Data Availability Statement

The datasets presented in this article are not readily available because the article used data obtained from the research activities of the Slovak Academic Association for Personnel Management SAAPM. Therefore, this data can be shared only with the consent of SAAPM. Requests to access the datasets should be directed to saapm.sk@gmail.com.

## Author Contributions

MB initiated the idea and worked on the analysis, worked on the relevant literature of the topic. PL and LL collected the data and performed the analyses and worked on the write up. All authors contributed to the article and approved the submitted version.

## Conflict of Interest

The authors declare that the research was conducted in the absence of any commercial or financial relationships that could be construed as a potential conflict of interest.

## Publisher’s Note

All claims expressed in this article are solely those of the authors and do not necessarily represent those of their affiliated organizations, or those of the publisher, the editors and the reviewers. Any product that may be evaluated in this article, or claim that may be made by its manufacturer, is not guaranteed or endorsed by the publisher.
